# Long-term efficacy of a continuity-of-care treatment model for patients with severe mental illness who transition from in-patient to out-patient services

**DOI:** 10.1192/bjp.2024.9

**Published:** 2024-04

**Authors:** Hagai Maoz, Rony Sabbag, Shlomo Mendlovic, Israel Krieger, Daphna Shefet, Ido Lurie

**Affiliations:** Shalvata Mental Health Center, Hod Hasharon, Israel; and Faculty of Medicine, Tel-Aviv University, Israel; Shalvata Mental Health Center, Hod Hasharon, Israel

**Keywords:** Psychotic disorders/schizophrenia, bipolar type 1 or 2 disorders, clinical outcomes measures, clinical trials, community mental health teams

## Abstract

**Background:**

Despite its significance, ensuring continuity of care demands substantial resources, which might not be readily accessible in many public healthcare systems. Studies indicate that continuity of care remains uncertain in numerous healthcare systems.

**Aims:**

This study aimed to assess the effectiveness of a continuity-of-care model for patients with severe mental illness (SMI), providing seamless treatment from discharge from a closed ward to subsequent psychiatric, psychological and rehabilitation services.

**Method:**

Data from patients discharged before (1 January to 31 December 2018) and after (1 June 2021 to 31 May 2022) full implementation of the model were analysed and compared in terms of average duration of hospital stay, emergency department visits within 90 days of discharge, readmission rate within a year post-discharge and initiation of rehabilitation process.

**Results:**

In the post-implementation period (*n* = 482), the average admission time significantly decreased from 30.51 ± 29.72 to 26.77 ± 27.89 days, compared with the pre-implementation period (*n* = 403) (*P* = 0.029). Emergency department visits within 90 days following discharge decreased from 38.70 to 26.35% of discharged patients (*P* < 0.001). The rate of readmission decreased from 50.9 to 44.0% (*P* = 0.041) for one readmission and from 28.3 to 22.0% (*P* = 0.032) for two readmissions in the year following discharge. Additionally, the proportion of patients entering formal rehabilitation increased from 7.94 to 12.03% (*P* = 0.044).

**Conclusions:**

This study highlights the effectiveness of a continuity-of-care model spearheaded by senior psychiatrists and involving paramedical personnel. These findings underscore the significant potential of the model to substantially enhance mental health services and outcomes. Moreover, they emphasise its relevance for patients, clinicians and policy makers.

Patients who experience severe mental illness (SMI), such as schizophrenia and bipolar disorder, require comprehensive therapeutic programmes.^1^ The chronic and fluctuating natural course of these illnesses necessitates an agile and responsive therapeutic system. Continuity of care and ongoing relationships with caregivers are of paramount importance in such cases.^2^ Discharge from hospital and readmission to ambulatory treatment is a critical transition period, and gaps between hospital and out-patient care have been associated with a significant increased risk of rapid readmission^3,4^ and suicide shortly after discharge,^5^ as well as a decrease in various measures of quality of life.^6^

Despite its importance, maintaining continuity of care often requires significant resources, which may not be readily available in all public healthcare systems. For example, recent evidence suggests a decline in continuity of care in the UK.^7^ In Israel, psychiatric hospitals typically maintain a complete separation between in-patient and out-patient systems, and most facilities have separate wards for different phases of treatment (closed wards, open wards and day care departments). Consequently, it is not uncommon for an in-patient with SMI to be transferred between two or even three treatment settings and caregiving teams within a short period of time. Similar to many mental health centres (MHCs), before implementing the presented model, our centre previously maintained a clear division between in-patient and out-patient treatment. Upon discharge from the in-patient wards, patients would undergo follow-ups within a specified period within the department until they transitioned to out-patient clinics, where they initiated psychiatric follow-up (or resumed follow-up if previously treated at the clinic). Treatment commonly involved regular psychiatric follow-ups and supportive therapy, predominantly facilitated by psychologists and social workers, conducted either individually or in group sessions for certain patients. Recognising the significance of continuity of care during and following a hospital stay, our MHC implemented a radical change several years ago. We transitioned from designated status-dependent departments (closed or open wards) to continuum wards that allow a full spectrum of in-patient care, including closed, open and day hospital stays. With this approach, the ward adapts to the patient's admission conditions based on the required level of supervision, facilitating long-term, in-depth care with a consistent caregiving team. Patients are allocated to wards based on their identification numbers, ensuring that in the event of readmission, each patient returns to the same ward and an equitable workload distribution is maintained among wards.

## The continuity-of-care project

In 2018, we initiated a pilot project for a comprehensive treatment system for patients with SMI, termed ‘the continuity project’, in one of the MHC's acute in-patient wards. A senior psychiatrist was appointed to serve as a ‘continuity physician’, who would divide his time between the in-patient ward and the out-patient clinic. Having familiarity with the patients on the ward, the continuity physician continued his out-patient care in the MHC's clinic, acting as the connecting link between in-patient and out-patient care. Over time, patients who successfully adapted to the community and deemed to have reduced risk of readmission were transitioned to the care of another psychiatrist from the same clinic. Following the success of the pilot project, we implemented this unique continuity model in all hospital wards, and established three ‘continuity divisions’ to serve all admitted patients with SMI within our MHC's catchment area (approximately 800 000 inhabitants). In a recent study,^8^ we demonstrated that maintaining this ‘continuity model’ significantly reduced the number of emergency department visits in the 30 days following hospital discharge, and lowered the readmission rates in the 30- and 90-day periods following discharge. However, shortly after implementation of the first stage of the model (positioning of a continuity physician), it became evident that addressing the complete psychosocial aspects of SMI treatment required more than just physicians. Therefore, occupational therapists, psychologists and art therapists from the original admitting wards integrated into the continuity division. This collaborative team was designed to encompass several therapeutic approaches tailored to meet each patient's specific needs. The duration of follow-up in the continuum clinic is usually set for a minimum of 1 year, aiming that after a year of clinical stability, the patient can continue psychiatric follow-up in regular out-patient care. The decision to conclude treatment at the continuum clinic is determined by the treating staff and remains flexible, contingent upon the patient's condition. In a sense, the continuum clinic allows for a higher frequency of visits compared with the period before the model implementation, providing a better opportunity to flexibly adjust the nature and frequency of meetings according to the patient's needs. Importantly, we did not increase the number of personnel (aside from the original physician), but instead redistributed some of the patient care responsibilities from in-patients wards to the ambulatory care setting. Consequently, every division within the hospital retained its original ward staff, and medical and paramedical professionals provide continuous ambulatory care after discharge and before potential readmission.

The biopsychosocial model^9^ emphasises that a crucial facet of comprehensive out-patient care involves providing patients access to rehabilitation services. Under Israeli law, psychiatric rehabilitation services are provided free to all eligible individuals. These services include supported housing, vocational rehabilitation, supported education and social and leisure activities,^10^ and clearly demand a certain level of patient motivation and stability, often extending beyond the time frame of acute hospital admission. Unfortunately, the time gap between patient discharge, out-patient admission and the transition to a new and unfamiliar staff may further delay this already complex process.

## Objectives

The main objective of the current study was to evaluate the effectiveness of the described continuity project, which involves continuous psychiatric, psychosocial and rehabilitation continuous care, on long-term outcomes. This evaluation encompassed several parameters, including duration of hospital stay, the time taken for initial psychiatric evaluation in the out-patient clinic, 1-year readmission rates and the utilisation of psychiatric rehabilitation services. Our hypotheses were that, compared with the period preceding the project's implementation, there would be a reduction in emergency department referrals in the following 90 days after discharge and readmission rates in the subsequent year, as well as an increase in the utilisation of community rehabilitation services for patients with SMI.

## Method

We conducted a retrospective evaluation of the continuity-of-care division, using various metrics to assess the quality of psychiatric care. These measures encompassed the mean duration of hospital stay, the time it took for patients to gain admission to clinical care after hospital discharge, emergency department visits within 90 days of discharge, the rate of readmission within 1 year post-discharge and the initiation of a rehabilitation process. We compared these measures between patients discharged from the wards before and after the implementation of the continuity model. Our analysis focused on patients discharged from two out of three divisions in the hospital, as the third division had not yet fully adopted the continuity model (mostly in terms of paramedical staff). The pilot ward initiated the continuity project in November 2018 by appointing the continuity physician. The other hospital wards appointed a continuity physician in 2020. During the same year, we introduced additional ‘continuity staff members’, including occupational therapists, psychologists and expressive art therapists for each division. To assess the impact of the continuity model, we examined two 1-year periods: one before model implementation (from 1 January to 31 December 2018) and another following its full implementation (from 1 June 2021 to 31 May 2022). We deliberately excluded periods during the COVID-19 pandemic to avoid any biases related to admission policies during that time.

For each patient, we set an ‘index admission’ (the patient's first hospital admission within the respective year) and analysed data on emergency department visits, the time interval between hospital discharge and the first out-patient clinic evaluation, and the number of readmissions in the subsequent year. The measurement of time lapse from hospital discharge until admission to the out-patient clinic applied to patients referred to the MHC clinic after discharge (most discharged patients had one or two follow-up appointments on the ward in the days or weeks following discharge, before continuing their psychiatric follow-up in the hospital's out-patient clinic).

The publication of the research data was approved by the Helsinki Internal Review Board (approval number SHA-23-15). Because of the retrospective nature of the study and the strict anonymity in data presentation, the need for patient informed consent was waived.

### Statistical analyses

Comprehensive data were collected concerning the relevant measures, taking great care to maintain strict anonymity to protect patient confidentiality. To compare quantitative data, we performed *t*-tests and *χ*^2^-tests as required between the two groups of patients, before and after the implementation of the continuity model. In the second stage, the bivariate Cox proportional hazards regression model was used to investigate potential factors that might have increased the risk for readmission, followed by the third stage, where a multivariate Cox proportional hazards regression model was used to obtain association between the variable (before/after implementation of the continuity model) and time to readmission, adjusting for variables that were significant (*P* < 0.1) at the second stage of analysis and the sociodemographic variables. All statistical analyses were carried out with SPSS version 25.0 for Windows (IBM Corp., New York, USA). All tests were two-sided with a significance level set at *P* < 0.05.

## Results

A total of 403 patients (*n* = 262 male, 65.01%) were discharged from the participating wards in the first period. Among them, 273 (67.74%) had a primary diagnosis of psychotic disorder, 72 (17.87%) an affective disorder, and 31 (7.69%) a severe personality disorder. Overall, 82 patients (20.34%) had a comorbid substance use disorder. In the second period, 482 (*n* = 304 male, 63.07%) were discharged. Most of the discharged patients had a diagnosis of psychotic disorder (*n* = 314, 65.14%), followed by affective disorders (*n* = 68, 14.10%) and severe personality disorders (*n* = 53, 11.00%). In this period, 110 patients (22.82%) had a comorbid substance use disorder. There were no statistically significant differences between the two groups in gender, primary psychiatric diagnosis or comorbid substance use disorder. All patients except for two were insured by one of four Israeli Health Maintenance Organisations (HMOs). A total of 298 patients (73.95%) continued their ambulatory follow-up in the hospital's out-patient clinic in the first period, and 368 patients (76.37%) continued their follow-up in the out-patient clinic in the second period (*χ*^2^ = 0.680, *P* = 0.41). Other patients typically continued their psychiatric follow-up in clinics of other HMOs or private clinics. In the second period, after the implementation of the project, 316 out of the 368 patients (85.86%) who continued follow-up in the hospital's out-patient clinic entered the continuity division (patients who did not suffer from an SMI that necessitated follow-up in an intensive therapeutic system pursued their treatment elsewhere). In this period, all patients were referred to psychiatric follow-up, 53 patients (16.26%) were referred for occupational therapy treatment in the out-patient clinic and 28 (8.59%) patients received psychological intervention, either from a clinical psychologist or expressive art therapist, in either a personal or group setting (importantly, some of the patients continued to receive psychological support and/or community rehabilitation services outside of the out-patient clinic service).

**Fig. 1:**
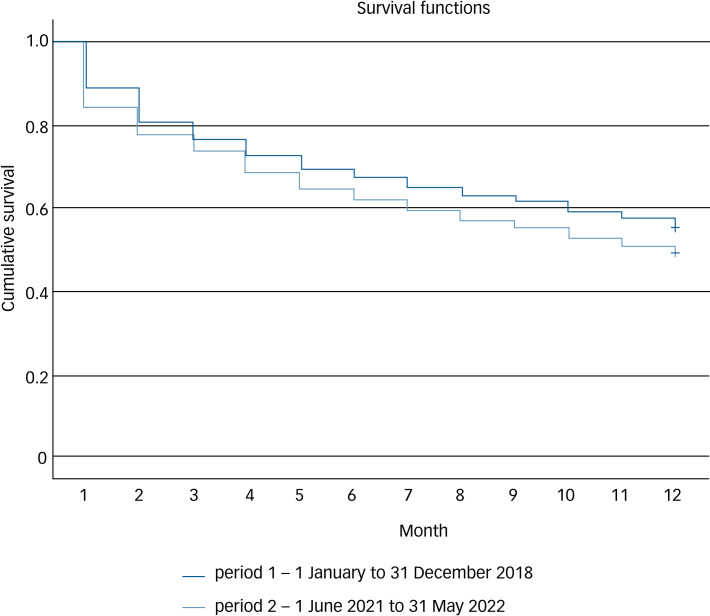
Rate of patients who were not readmitted to hospital in the first year after discharge.

The average in-patient admission time was 30.51 ± 29.72 days in the first period and 26.77 ± 27.89 days in the second period (*t* = 1.89, *P* = 0.029). The average duration for the initial psychiatric evaluation in the out-patient clinic following discharge from the in-patient setting decreased from 26.41 ± 7.14 to 23.19 ± 7.42 days; however, this difference was not statistically significant (*t* = 1.58, *P* = 0.071). The rate of emergency department visits within 90 days following discharge significantly decreased from 156 (38.70%) to 127 (26.35%) (*χ*^2^ = 15.418, *P* < 0.001). As can be seen in Table 1, there was a significant decrease in the number of patients readmitted once or twice in the year following discharge, from 50.9 to 44.0% (*χ*^2^ = 4.181, *P* = 0.041) for one readmission and from 28.3 to 22.0% (*χ*^2^ = 4.658, *P* = 0.032) for two readmissions. However, there was no statistically significant decrease in the readmission rate for patients requiring three or more admissions in a year. Figure 1 illustrates the rate of patients not readmitted in the first year after discharge during the two time periods.

**Table 1:** Number of patients readmitted to hospital following the first year after discharge, before and after the implementation of the continuity-of-care model

	Period 1 (*n* = 403)^a^	Period 2 (*n* = 482)^b^	Statistics	*P*-value
Admission time, mean ± s.d. (days)	30.51 ± 29.72	26.77 ± 27.89	*t* = 1.89	**0.029**
Time for initial psychiatric evaluation in the out-patient clinic (days)	26.41 ± 7.14	23.19 ± 7.42	*t* = 1.58	0.071
Emergency department visit during 90 days after discharge	156 (38.70%)	127 (26.35%)	*χ*^2^ = 15.418	**<0**.**001**
At least one readmission	205 (50.9%)	212 (44.0%)	*χ*^2^ = 4.181	**0**.**041**
At least two readmissions	114 (28.3%)	106 (22.0%)	*χ*^2^ = 4.658	**0**.**032**
At least three readmissions	71 (17.6%)	67 (13.9%)	*χ*^2^ = 2.305	0.129
Four or more readmissions	19 (4.7%)	18 (3.7%)	*χ*^2^ = 0.526	0.468
Patients entering rehabilitation process	32 (7.94%)	58 (12.03%)	*χ*^2^ = 4.024	**0**.**044**

Bold indicates significance at *P* < 0.05.

a. Period 1: 1 January to 31 December 2018.

b. Period 2: 1 June 2021 to 31 May 2022.

Finally, we conducted a comparison of the rate of patients with SMI who underwent rehabilitation committee processes, either during hospital stay or following discharge, between the two time periods. The proportion of patients who entered the rehabilitation process after the implementation of the continuity model (12.03%) was significantly higher than before its adoption (7.94%) (*χ*^2^ = 4.024, *P* = 0.044).

## Discussion

The primary objective of the current study was to assess the effectiveness of our MHC's in-patient to out-patient continuity-of-care model by utilising various quality-of-care measurements. The central structure of this model facilitates the seamless integration of in-patient and out-patient settings, allowing for the provision of sustained treatment to patients with SMI over extended periods. The primary objective of this model is to improve treatment outcomes by bridging the gaps that occur during the transition from discharge to entering therapy in out-patient services, as well as by minimising changes in the providing team. The main findings of the study indicate a reduction in the length of stay on the in-patient ward and a decrease in the average time required for the initial psychiatric evaluation in the out-patient clinic (which, in the current model, is performed usually by a senior psychiatrist who is already familiar with the patient from their stay on the in-patient ward). More notably, there was a significant decrease in the rate of emergency department visits within 90 days following discharge, along with a decrease in readmission rate over the subsequent year after discharge, particularly for patients who required one or two readmissions within that time frame.

Although continuity of care in psychiatry is frequently regarded as a fundamental element of modern healthcare provision, there is no consensus regarding its precise definition.^3,11,12^ Literature on continuity often centres on the interval spanning from hospital discharge to out-patient admission, evaluating its quality based on clinical outcomes such as the risk of readmission,^3,4^ symptom severity,^6,11,13^ social functioning,^13–15^ suicide risk^5^ and the post-discharge quality of life for patients.^6,11^ The findings of the current study add to the limited existing knowledge regarding the fundamental aspects of the treatment sequence. They illustrate how a modification in the treatment system, without necessarily requiring additional workforce, can result in a significant improvement in certain conventional outcome measures for patients with SMI. The significant reduction observed in the rate of emergency department visits and readmissions in the year following discharge from the in-patient ward is probably indictive of improved quality of care for patients with SMI. This finding aligns with previous studies demonstrating that maintaining a continuous treatment programme decreases the relative risk of readmission for patients with SMI.^15–17^ Notably, the fact that the finding of a significant decrease in the rate of patients readmitted once or twice during the following year after discharge was not observed for patients requiring three or more admissions, likely suggests that the current model may not adequately serve as an anchor for the most severely affected patients, who require more intensive resources such as long-term facilities. Beyond what appears to be an enhancement in comprehensive care for patients with SMI, the combination of reduced in-patient ward stays and decreased readmission rates suggests that emergency department and ward staff can allocate their resources to other patients requiring intensive attention. We assume that the decline in referral rates to the emergency department within months following discharge from the in-patient setting is attributed to establishment of a more direct and swift connection with a medical team familiar with the patients from their hospital stay. Additionally, we hypothesise that a notable decrease in readmission rates is a result of the presence of a transitional infrastructure comprising pre-existing familiarity of the patient with both a psychiatrist and the paramedical team. This configuration contributes significantly to fortifying the support system for patients during the post-discharge period and at the initial stages of the rehabilitation process, where there is a heightened potential for psychological regression. The substantial assistance provided by the paramedical team plays a pivotal role in facilitating community reintegration. It is important to highlight the benefits of the current continuity-of-care model in increasing the rate of patients with SMI who enter the rehabilitation process. In recent decades, the deinstitutionalisation movement has gained momentum, underscoring the importance of shifting the focus of psychiatric treatment from hospital settings to the community.^18^ Approximately 3% of patients with SMI in Israel are annually referred to a ‘rehabilitation committee’ to access new rehabilitation services.^19^ As demonstrated, our centre exhibited higher rate of referrals to the rehabilitation committee even before the model's implementation (7.94% of discharged patients per year). However, these referral rates increased even further, to 12.03%. This increase likely indicates that the continuity model fosters awareness of rehabilitation from the early stages of treatment and enables its implementation when the patient is stabilised.

In certain respects, the model presented here draws inspiration from other intensive community treatment models that have demonstrated reduced readmission rates. For instance, research has indicated the effectiveness of a brief critical time intervention following discharge,^20^ or the assertive community treatment model (ACT) in decreasing hospital admission rates, particularly involuntary admissions.^21^ Nevertheless, the advantages of these models remain a topic of controversy, concerning both clinical efficacy and cost-effectiveness.^22^ Unlike ACT, the current model highlights a treatment continuum, facilitating a smoother integration between in-patient and community services. Importantly, we primarily utilised existing personnel and did not significantly expand the treatment team. It is worth emphasising that implementing the continuity-of-care model in our centre was relatively straightforward. This is primarily because most of the patients discharged are referred to our ambulatory unit, which serves patients from all Israeli HMOs. Furthermore, the catchment area of our hospital is relatively small. Consequently, it is possible that implementing such a model would be considerably more complex in larger catchment areas with intricate relationships between the hospital's out-patient clinics and community HMOs. Although the hospital staff adapted smoothly to the presented continuity-of-care model, it is crucial to investigate over time whether this model also leads to an increase in therapist burnout, which is highly prevalent in professionals who treat patients with SMI.^23^

The main limitation of the current study is that the model was implemented in a specific hospital with a unique history of community service. The study relies on data exclusively from this centre, and is not a multi-centre study. The specificity of the implementation site emphasises the importance of individualised factors, making it challenging to discern the extent to which the model's success is attributed to the structural change in the hospitalisation system or to the personnel themselves. This may hamper the ability to generalise the model's outcomes to other MHCs, affecting external validity. To enhance generalisability, utilising a larger data-set on a national level would enable more robust conclusions. However, even with a broader data-set, it remains complex to extend these findings to other nations with distinct mental healthcare systems. Furthermore, a notable limitation is the absence of clinical parameters such as the Positive and Negative Syndrome Scale or Outcome Questionnaire 45, which are essential for assessing patients’ quality of life. Consequently, it is debatable whether the differences observed in this study genuinely reflect improvement in patient care and quality of life.

In conclusion, this study underscores the effectiveness of a continuity-of-care model, which revolves around a ‘continuity team’ led by senior psychiatrists and comprises paramedical personnel, including occupational therapists, psychologists and expressive art therapists, for patients with SMI. The model's foundation lies in the belief that cultivating an ongoing therapeutic relationship fosters trust, knowledge and stability, ultimately enhancing treatment outcomes. These findings emphasise the substantial potential of the model to notably improve mental health services and outcomes, and of its relevance for patients, clinicians and policy makers.

## Data Availability

The data that support the findings of this study are available on request from the corresponding author, H.M. The data are not publicly available due to their containing information that could compromise the privacy of research participants.

## References

[1] GBD 2019 Mental Disorders Collaborators. Global, regional, and national burden of 12 mental disorders in 204 countries and territories, 1990–2019: a systematic analysis for the Global Burden of Disease Study 2019. Lancet Psychiatry 2022; 9(2): 137–50. 35026139 10.1016/S2215-0366(21)00395-3 PMC8776563

[2] Crawford MJ, de Jonge E, Freeman GK, Weaver T. Providing continuity of care for people with severe mental illness a narrative review. Soc Psychiatry Psychiatr Epidemiol 2004; 39(4): 265–72. 15085327 10.1007/s00127-004-0732-x

[3] Puntis SR, Rugkasa J, Burns T. The association between continuity of care and readmission to hospital in patients with severe psychosis. Soc Psychiatry Psychiatr Epidemiol 2016; 51(12): 1633–43. 27783129 10.1007/s00127-016-1287-3 PMC5131080

[4] Killaspy H, Banerjee S, King M, Lloyd M. Prospective controlled study of psychiatric out-patient non-attendance. Characteristics and outcome. Br J Psychiatry 2000; 176: 160–5. 10755054 10.1192/bjp.176.2.160

[5] Haglund A, Lysell H, Larsson H, Lichtenstein P, Runeson B. Suicide immediately after discharge from psychiatric inpatient care: a cohort study of nearly 2.9 million discharges. J Clin Psychiatry 2019; 80(2): 18m12172. 10.4088/JCP.18m12172 30758922

[6] Catty J, White S, Clement S, Cowan N, Geyer C, Harvey K, et al. Continuity of care for people with psychotic illness: its relationship to clinical and social functioning. Int J Soc Psychiatry 2013; 59(1): 5–17. 21948559 10.1177/0020764011421440

[7] Macdonald A, Adamis D, Craig T, Murray R. Continuity of care and clinical outcomes in the community for people with severe mental illness. Br J Psychiatry 2019; 214(5): 273–8. 31012407 10.1192/bjp.2018.261

[8] Maoz H, Sabbag R, Krieger I, Mendlovic S, Shefet D. The impact of a continuity-of-care model from hospitalization to outpatient clinic for patients with severe mental illness. Psychiatr Serv 2023; 74(5): 551–4. 36196530 10.1176/appi.ps.202100508

[9] Engel GL. The need for a new medical model: a challenge for biomedicine. Science 1977; 196(4286): 129–36. 847460 10.1126/science.847460

[10] The Labour, Welfare and Health Committee. *The Rehabilitation of the Mentally Disabled Act. The Israeli Knessat 2000 (Parliament) (in Hebrew)*. The Knessat (https://www.health.gov.il/LegislationLibrary/Nefesh35.pdf).

[11] Adair CE, McDougall GM, Mitton CR, Joyce AS, Wild TC, Gordon A, et al. Continuity of care and health outcomes among persons with severe mental illness. Psychiatr Serv 2005; 56(9): 1061–9. 16148318 10.1176/appi.ps.56.9.1061

[12] Johnson S, Prosser D, Bindman J, Szmukler G. Continuity of care for the severely mentally ill: concepts and measures. Soc Psychiatry Psychiatr Epidemiol 1997; 32(3): 137–42. 9130865 10.1007/BF00794612

[13] Brekke JS, Ansel M, Long J, Slade E, Weinstein M. Intensity and continuity of services and functional outcomes in the rehabilitation of persons with schizophrenia. Psychiatr Serv 1999; 50(2): 248–56. 10030485 10.1176/ps.50.2.248

[14] Sytema S, Burgess P. Continuity of care and readmission in two service systems: a comparative Victorian and Groningen case-register study. Acta Psychiatr Scand 1999; 100(3): 212–9. 10493088 10.1111/j.1600-0447.1999.tb10848.x

[15] Olfson M, Mechanic D, Boyer CA, Hansell S. Linking inpatients with schizophrenia to outpatient care. Psychiatr Serv 1998; 49(7): 911–7. 9661225 10.1176/ps.49.7.911

[16] Bindman J, Johnson S, Szmukler G, Wright S, Kuipers E, Thornicroft G, et al. Continuity of care and clinical outcome: a prospective cohort study. Soc Psychiatry Psychiatr Epidemiol 2000; 35(6): 242–7. 10939422 10.1007/s001270050234

[17] Grinshpoon A, Lerner Y, Hornik-Lurie T, Zilber N, Ponizovsky AM. Post-discharge contact with mental health clinics and psychiatric readmission: a 6-month follow-up study. Isr J Psychiatry Relat Sci 2011; 48(4): 262–7. 22572089

[18] Fakhoury W. Deinstitutionalization and reinstitutionalization: major changes in the provision of mental healthcare. Psychiatry 2007; 6(8): 313–6.

[19] Bergman-Levy T. *Mental Health in Israel: Statistical Abstract 2018*. State of Israel, Ministry of Health, 2020 (https://www.gov.il/BlobFolder/reports/mtl-yearbook-2020/he/files_publications_units_mental_health_MentalHealth2020.pdf).

[20] Shaffer SL, Hutchison SL, Ayers AM, Goldberg RW, Herman D, Duch DA, et al. Brief critical time intervention to reduce psychiatric rehospitalization. Psychiatr Serv 2015; 66(11): 1155–61. 26234327 10.1176/appi.ps.201400362

[21] Phillips SD, Burns BJ, Edgar ER, Mueser KT, Linkins KW, Rosenheck RA, et al. Moving assertive community treatment into standard practice. Psychiatr Serv 2001; 52(6): 771–9. 11376224 10.1176/appi.ps.52.6.771

[22] Burns T. The rise and fall of assertive community treatment? Int Rev Psychiatry 2010; 22(2): 130–7. 20504053 10.3109/09540261003661841

[23] Bykov KV, Zrazhevskaya IA, Topka EO, Peshkin VN, Dobrovolsky AP, Isaev RN, et al. Prevalence of burnout among psychiatrists: a systematic review and meta-analysis. J Affect Disord 2022; 308: 47–64. 35398112 10.1016/j.jad.2022.04.005

